# Autophagy suppression of trophoblast cells induces pregnancy loss by activating decidual NK cytotoxicity and inhibiting trophoblast invasion

**DOI:** 10.1186/s12964-020-00579-w

**Published:** 2020-05-12

**Authors:** Hai-Xia Tan, Shao-Liang Yang, Ming-Qing Li, Hai-Yan Wang

**Affiliations:** 1 Department of Gynecology of Integrated Traditional Chinese and Western Medicine, Hospital of Obstetrics and Gynecology, Fudan University, Shen Yang Road 128, Shanghai, 200090 People’s Republic of China; 2 NHC Key Lab of Reproduction Regulation (Shanghai Institute of Planned Parenthood Research), Hospital of Obstetrics and Gynecology, Fudan University, Pingliang Road, Shanghai, 200080 People’s Republic of China; 3 Laboratory for Reproductive Immunology, Hospital of Obstetrics and Gynecology, Fudan University, Shanghai, 200080 People’s Republic of China; 4 Shanghai Key Laboratory of Female Reproductive Endocrine Related Diseases, Shanghai, 200080 People’s Republic of China

**Keywords:** RSA, Trophoblast autophagy, NK cell, IGF-2, PEG10

## Abstract

**Background:**

The crosstalk between trophoblast cells and decidual NK cells plays an important role in the establishment and maintenance of normal pregnancy. Recent studies reported that autophagy can induce immune tolerance at the maternal fetal interface, while the mechanism remains unclear.

**Methods:**

Autophagy levels in the villi of normal and recurrent spontaneous abortion (RSA) patients were detected by transmission electron microscopy. After co-cultured with trophoblast cells pretreated with 3-MA or rapamycin, NK cells were collected and the expression of killer receptors was detected by flow cytometry (FCM). The invasiveness of trophoblasts was tested by Cell invasion assay.

**Results:**

Compared with elective pregnancy termination patients, the level of autophagy in the villi of RSA patients was significantly decreased. Inducing the autophagy level in trophoblast cells with rapamycin could significantly inhibit the cytotoxicity of NK cells in the co-culture system, and supplement of IGF-2 could rectify this effect. Meanwhile, autophagy suppression of trophoblasts reduced the level of Paternally Expressed Gene 10 (PEG10), leading to the impairment of trophoblast cell invasion. In addition, NK cells educated by autophagy-inhibited trophoblasts further decreased the proliferation and invasiveness of trophoblasts. In pregnant mice model, injection with 3-MA promoted the cytotoxicity of uterine NK cells, and increased the embryo absorption rate.

**Conclusion:**

Autophagy suppression of trophoblasts increase the cytotoxicity of NK cells and damage the trophoblasts invasion possibly by targeting IGF-2 and PEG10, respectively, which ultimately leads to miscarriage.

Video Abstarct

## Background

According to the latest ESHRE guidelines, recurrent spontaneous abortion (RSA) is defined as two or more consecutive miscarriages before 24 week, occurring in about 1–2% of pregnant women [1]. The etiology of RSA is varied, mainly involving genetic factors, immune factors, endocrine dysfunction, infections, reproductive tract anatomic abnormalities, etc. [2–4]. But the cause is still unclear in a large proportion of patients. Among the patients with unknown etiology, the immune factors are of great importance [5].

Decidual natural killer cells (dNK) account for more than 70% of immune cells at fetal-maternal interface in early pregnancy [6], and they were found to perform various functions in the process of decidualization, uterine vascular remodeling and immune tolerance inducing [7, 8]. The aberration of proportion or function of dNK cells are closely related to the occurrence of RSA. In addition, there exists cross-talk and interaction between NK cells and trophoblast cells. Trophoblasts can transmit signal to dNK cells by direct contact, or regulate NK cells function by expressing and secreting a variety of cytokines and chemokines [9–11]. In turn, it has been reported that IL-8 secreted by dNK cells plays an important role in placenta formation and trophoblast cell invasion [12]. The abnormal interaction between dNK and trophoblasts is associated with failure of pregnancy and abortion.

Autophagy is a catalytic process that maintains cell homeostasis in eukaryotic cells, including the degradation of damaged macromolecules and cytoplasmic components of organelles [13, 14]. Autophagy usually refers to macroautophagy, which is characterized by autophagosomes of double-membrane vesicles [15]. At present, autophagy is mainly considered as an effector and regulator of adaptive immunity, and many studies have demonstrated its role in intracellular pathogen responses [16, 17]. When cells are under hypoxia, nutrient deprivation, endoplasmic reticulum stress, mitochondrial damage, immune signals or inflammatory stimulation, autophagy was induced through various signaling pathways to maintain the normal metabolism of cells [18]. In particular, the autophagy inhibition of trophoblast induces IL-1β secretion, thus triggering the over activated inflammatory response at the maternal-fetal interface, which may be a pathogenic mechanism of RSA induced by anti-phospholipid antibody syndrome [19]. However, the specific roles and mechanisms of autophagy in regulating the crosstalk between trophoblasts and decidual NK cells still need to be elucidated.

Trophoblast cells shares many characteristics with tumor cells in inducing and maintaining immune tolerance, especially in inducing immune tolerance of NK cells. Some researchers have found that the induction of autophagy in breast cancer cells under hypoxic conditions can effectively degrade granzyme B secreted by NK cells, thereby reducing the sensitivity of tumor cells to NK cell killing [20]. In addition, autophagy activation has been found to damage NK cell-mediated cytotoxicity in melanoma, non-small cell lung cancer and liver cancer [21–23].

Despite the fact that there exist many similarities between tumors and fetuses or allograft rejection and abortion [24], but the role of trophoblasts autophagy in maternal-fetal immune regulation remains unclear. Therefore, the aim of this study was to investigate the role of autophagy in trophoblast cells in regulating the cytotoxicity of decidual NK cells, and to explore the mechanisms of cross-talk between dNK cells and trophoblast cells in miscarriage in vitro and in vivo.

## Methods

### Patients and samples collection

The study protocol was approved by the Human Ethics Committee of Obstetrics and Gynecology Hospital, Fudan University, and all participants provided written informed consent. The elective pregnancy termination patients and RSA patients included in this study were at the age of 20–38 years and the gestational age was 7–9 weeks. All of them were from the Obstetrics and Gynecology Hospital of Fudan University between May 2018 and August 2019. The decidual (*n* = 40) and villi tissues (*n* = 14) were from women with normal pregnancy. Normal pregnancy patients had no history of spontaneous abortion, stillbirth and other adverse pregnancies. Blood routine and vaginal discharge tests were normal. There was no vaginal bleeding, abdominal pain, fever, pathogen infection or obvious local inflammation in all of the patients. The decidual tissues were stored in ice-cold DMEM / F-12 (HyClone, USA) under sterile conditions and transported to the lab within 1 h after surgery. Then, the decidual immune cells (DIC) were isolated and cultured. Villi tissues were immersed in electron microscopic fluid for subsequent test.

### dNK cells isolation and cell culture

The decidual tissues were washed in PBS (HyClone), then cut into 1 mm^3^ pieces and digested with 20% type IV collagenase (0.1%; Sigma-Aldrich, USA) and 5% DNA enzyme (3000 IU, Sigma, Germany) at 37 °C for 30 min. The tissue fragments were filtered through sieves (pore size: 100, 300 and 400 mesh), centrifuged at 1300 rpm for 10 min, and the supernatant was discarded. 20, 40, and 60% Percoll (Amersham, USA) were prepared, and then the lower layer DIC was recovered by density gradient centrifugation at 2500 rpm for 30 min. DIC was cultured overnight in RPMI-1640 medium (HyClone) containing 10% fetal bovine serum (FBS, Gibco, USA). According to the manufacturer’s instructions, NK cells were negatively selected from DIC using human NK cell separation kits (MACS, Miltenyi Biotec, Germany). The dNK purity measured by FCM was over 90%.

NK cells were cultured in RPMI-1640 (HyClone, USA) containing 10% FBS (Gibco) and 1% penicillin-streptomycin solution (HyClone, USA). Furthermore, IL-2 (20 ng/μl), IL-15 (20 ng/μl) and IGF-2 (50 ng/μl) were supplemented according to different experiments. HTR-8/SVneo cell lines were cultured in DMEM/F12 (HyClone) containing 10% FBS (Gibco) and 1% penicillin-streptomycin solution (HyClone).

### Co-culture of dNK cells and HTR-8/SVneo

HTR-8 / SVneo was pretreated with or without rapamycin (2 μM, Sigma, USA) for 48 h in a 24-well plate (Corning, USA) and then co-cultured with dNK cells. In addition, HTR-8 / SVneo was pretreated with or without 3-MA (10 mM, Sigma, USA) for 24 h and then co-cultured with dNK cells. The ratio of dNK cells (2 × 10^5^ cells/well) to HTR-8 / SVneo (1 × 10^5^ cells/well) was 2:1. After 48 h of co-culture, all suspended cells in the co-culture system were collected for subsequent experiments.

### Transmission electron microscopy

The villi of RSA patients and elective pregnancy termination patients were collected, and the tissues’ volume were generally not more than 1mm^3^. Fresh tissues were quickly placed into the electron microscope fixative (Servicebio), fixed at 4 °C for 2–4 h, and post-fixed in 1% osmium acid for 2 h. The samples were dehydrated in a series of gradient concentration alcohols, permeated overnight with a mixture of acetone and 812 embedding agent (SPI) (2:1), then embedded in pure 812 embedding agent and polymerized in a 60 °C oven for 48 h. Samples were cut into 60-80 nm ultrathin sections by using ultrathin slicing machine (Leica UC7). The sections were double stained with uranium and lead (2% uranyl acetate saturated alcohol solution, lead citrate) and dried overnight at room temperature. Finally, observed under a transmission electron microscope (HITACHI, HT7700), collected images for analysis.

### Lentiviral transfection

HTR-8 / SVneo cells were seeded in a six-well plate (5 × 10^4^ cells/ml). After confluence reached 30%, the cells were infected with ATG5 silencing lentivirus (ATG5-RNAi)/PEG10 overexpression lentivirus (PEG10^over^), and their corresponding negative control virus (NC) (all from Genechem Co., LTD.). According to the manufacturer’s instructions, the optimal transfection condition of MOI was 80% of infected cells in the best time. Calculated the required virus volume, after 12 h of the cells were infected, the medium was changed and continued to culture. The transfection efficiency was observed under a fluorescence microscope at 48 h and 72 h, and subsequently screened for 1 week using 1 μg/ml puromycin (Genechem Co., LTD.).

### qRT-PCR

The cells or mouse placental tissues were collected, and total RNA was extracted by RNAiso Plus reagent (TaKaRa Biotechnology). According to the manufacturer’s instructions, 1000 ng of total RNA was reverse transcribed into cDNA with a reverse transcription kit (TaKaRa Biotechnology). Subsequently, detection was carried out on a real-time PCR instrument (ABI QuantStudio 6 Flex, USA). Reaction system (10 μl): 5 μl TB Green Premix Ex TaqTM II, 0.2 μl ROX Reference Dye II, 1 μl cDNA, 0.4 μl Forward Primer, 0.4 μl Reverse Primer and 3 μl RNase Free dH2O. Primers were listed in Table 1. Reaction conditions (40 cycles): denaturation (95 °C 30s), annealing (95°C5s) and elongation (60 °C 34 s). Finally, the infection efficiency of lentivirus siATG5, PEG10^over^ and the expression of related molecules were analyzed using the 2^-ΔΔCT^ method.

**Table 1:** Related primer sequences

Genes	Primer sequences (5′–3′)	Reverse primer (5′–3′)
GAPDH	GTATCGTGGAAGGACTCATGAC	ACCACCTTCTTGATGTCATCAT
ATG5	GATGGGATTGCAAAATGACAGA	GAAAGGTCTTTCAGTCGTTGTC
MAP1LC3B	TTATTCGAGAGCAGCATCCAACC	CCGTTCACCAACAGGAAGAAGG
BECN1	ATCTAAGGAGCTGCCGTTATAC	CTCCTCAGAGTTAAACTGGGTT
IL7R	AGGCTTCTGGAGTGAATGGAGTCC	CCAAGATGACCAACAGAGCGACAG
TRIM22	CATCACTGCAAAGATCAAGGAG	TGACCTCTTTGACTCTCTCAAC
DDIT3	GAGAATGAAAGGAAAGTGGCAC	ATTCACCATTCGGTCAATCAGA
IL13RA2	AATTTGGAGTGAGTGGAGTGAT	CAAATGGTAGCCAGAAACGTAG
SERPINB2	CCCATGACTCCAGAGAACTTTA	CTGCAAAATCGCATCAGGATAA
MMP1	AGATTCTACATGCGCACAAATC	CCTTTGAAAAACCGGACTTCAT
MYC	CGACGAGACCTTCATCAAAAAC	CTTCTCTGAGACGAGCTTGG
NES	TTGAAAAAGAGACTCAACAGCG	AAGATTTTACTGCCTCTACGCT
HAND2	AACTCTCCAAAATCAAGACCCT	GATTTCGTTCAGCTCCTTCTTC
IGF2	CTGGAGACGTACTGTGCTAC	CATATTGGAAGAACTTGCCCAC
CFH	GTGACTTACACTTGTGCAACAT	GGGCTCCTACATTGATAACGTA
MUC1	CACAGTGCTTACAGTTGTTACG	TGGTCATACTCACAGCATTCTT
CD82	ACAAGAGCAGTTTCATCTCTGT	CTTGCCCATGTTGAAGTAGAAG
TIMP3	AAGCAGATGAAGATGTACCGAG	GTACTTGTTGACCTCCAGCTTA
PLAC8	TGCAGCTGATATGAATGAATGC	TACAATGAGGACAGCAAAGAGT
PEG10	GATCTTCATGGAAAAGAGCACC	CATCATGAAAGCTGGGTAGTTG

Table 1. Related primer sequences.

### Transcriptome sequencing

HTR-8/SVneo cells transfected with ATG5-RNAi (*n* = 3) and NC (n = 3) were added to RNAiso Plus reagent (TaKaRa), hereafter submitted to Shanghai Litzchi Biosystems (LITCHI BIO, Shanghai, China) for subsequent RNA-seq analysis. Specific processes include RNA extraction, RNA sample quality inspection, library construction, library purification, library detection, library quantification, sequencing cluster generation, and sequencing on the Hiseq 4000 platform. FastQC software (V0.10.1) was used to control the quality of the offboard data, and then DESeq2 (V1.6.3) of Bioconductor software package was used to analyze and screen the differential genes. The differentially expressed gene standards were as follows: expression amount fold difference threshold |logFC| > 1; expression difference significance threshold *P*-value < 0.05. The results of sequencing were analyzed and produced a related thermograms using pheatmap Version 1.0.8 in R3.4.1. In addition, based on the STRING database, the protein interaction relationship between the differential genes and the interest genes (NK function-related genes, invasion-related genes, autophagy-related genes) were predicted, and then the network maps were constructed via the Cytoscape software.

### Protein extraction and western blot

After the cells were washed with PBS, lysate (RIPA: 100 XPMSF = 100:1) (Beyotime, china) was added, and the cells were separated by cell scraper after 30 min on ice, centrifuged at 12,000 rpm for 30 min at 4 °C. Then the supernatant was collected and the protein concentration was determined by BCA Protein Assay Kit (Beyotime, China). 1/4 of 5 × SDS-PAGE (Beyotime, china) was added according to the protein volume, boiled at 99 °C for 10 min, and then store at − 80 °C. In 12.5% SDS-PAGE (Epizyme, Shanghai, China), the total protein (30 μg / pore) was electrophoretic and transferred to the PVDF membrane (Millipore, USA). The membrane was sealed at room temperature for 2 h with 5% skim milk, and then washed 3 times for 15 min each using TBST (Sangon Biotech). Incubated with the primary antibodies against P62 (1:1000; Cell Signaling Technology,USA), LC3B (1:1000; Cell Signaling Technology, USA), ATG5 (1:3000; Abcam, Cambridge, UK), PEG10 (1:5000; Abcam, Cambridge, UK), GAPDH (1:1000; Cell Signaling Technology,USA), α-Tubulin (1:2000; Proteintech,USA) overnight at 4 °C. Thereafter, the membranes were washed again 3 times with TBST, then incubated with horseradish peroxidase (HRP) conjugated goat-anti-rabbit IgG secondary antibody (1:3000; Cell Signaling Technology, USA) for 1 h at room temperature. Finally, the membrane was washed 3 times and subjected to chemiluminescence treatment using an ECL Detection Kit (Millipore, USA).

### Flow cytometry (FCM)

dNK cells co-cultured with HTR-8 / SVneo were collected from 24-well plates and centrifuged at 1300 rpm for 8 min at room. According to the recommended dose, dNK cells were stained with fluorescent dye-conjugated antibody of human antigen at 4 °C for 30 min, including APC-conjugated anti-human CD56, FITC-conjugated anti-human CD16, APC/CY7-conjugated anti-human CD107a, PE-conjugated anti-human NKG2D/ Granzyme B, PE/CY7 -conjugated anti-human NKP30, and BV421-conjugated anti-human NKP46/IFN-γ (all from BioLegend), or the isotype control. Among them, the intracellular molecules (Granzyme B, IFN-γ) were added to FOXP3 Fix/Perm Buffer (4×) (BioLegend) to fix and break the membrane, then stained with antibody for 30 min. After that, the cells were washed twice with PBS and resuspended. The samples were tested using a CyAN ADP analyzer (Beckman Coulter, USA) and analyzed by FlowJo software (TreeStar, USA). In isotype matched controls, the statistically labeled positive cells should be less than 5%.

Mice NK cells were used PE-conjugated anti-mouse NK1.1, APC/Cy7-conjugated anti-mouse CD3, PerCP/Cy5.5-conjugated anti-mouse CD16, FITC-conjugated anti-mouse NKG2D, APC-conjugated anti-mouse NKP46/IFN-γ, BV421-conjugated anti-mouse CD107a and FITC-conjugated anti-human/mouse Granzyme B (all from BioLegend) to stain. The samples were then analyzed by flow cytometry.

### Immunohistochemistry

The paraffin sections of human villi (5 μ m) were dehydrated in graded ethanol, next the endogenous peroxidase was removed with 3% hydrogen peroxide and incubated with 5% BSA at room temperature for 1 h. After that, the samples were incubated with rabbit anti--human IGF-2 (1:200; abcam); rabbit anti--human PEG10 (1:500; abcam) or rabbit IgG isotypes at 4 °C overnight. After washing with PBS for three times, the sections were incubated with HRP-labeled secondary antibody at room temperature, reacted with 3,3-diaminobiphenylamine (DAB), and finally counterstained with hematoxylin.

### Cell invasion assay

Matrigel (BD Bioscience) was diluted at a ratio of 1:8, and 35 μL was added to the transwell upper chamber (8 μm, Corning). The transwell chambers were placed in a 24-well plate for overnight stay at 4 °C. 200 μl (HTR-8 / SVneo, 2 × 10^4^ cells/well) DMEM/F12 suspension without FBS was added to the upper chamber, and 600 μl DMEM / F12 containing 10%FBS was added to the lower chamber. According to different experimental requirements, 3-MA was added or not in the upper chamber, dNK cells (1 × 10^5^ cells/well) were added or not in the lower chamber. The cells were cultured for 48 h at 37 °C, 5% carbon dioxide incubator. The 24-well plate was removed, and the upper chamber medium and non-penetrating cells were gently wiped off with a cotton swab, PBS washed 3 times, fixed with 4% paraformaldehyde for 30 min, and crystal violet stained for 20 min. Thereafter, random photographs were taken under an inverted microscope (× 100), and each chamber counted 5 visual fields. The number of invaded cells was counted by ImageJ software.

### The cell-counting kit-8 (CCK-8) assay

CCK-8 assay (Dojindo, Tokyo, Japan) was used to detect the proliferation ability of HTR-8/SVneo cells after co-culture. The cells were seeded in 96-well plates (5 × 10^3^ cells/well) for 0 h, 24 h, 48 h and 72 h, respectively. Then, 10 μl CCK-8 solution was added to each well and cultured for 2 h, the absorbance value at 450 nm was measured by microplate reader. Six parallel holes were set for each experiment and repeated three times.

### In vivo experiments (mice)

All mice used in the experiment were C57BL/6 J strain (Shanghai jiesijie experimental animal Co., Ltd.) and were raised in an SPF experimental animal facility. 8-week-old female mice and 8-week-old male mice were caged at 2:1, and the pregnancy was confirmed on the day when the vaginal plug was seen (0.5 days). Pregnant mice were randomly divided into control group and experimental group. The experimental group was intraperitoneally injected with 3-MA (100 mg/kg /time, Sigma) at day 0.5, day 4.5, day 10.5, and the control group was injected with saline of equal volume at the same days. At day 8.5, the same amounts of pregnant mice in the control group and the experimental group were killed, and the number of embryo implantation and embryo absorption in the two groups were recorded respectively. The decidua cells of mice uterus were obtained by shredding, digesting, filtering and centrifuging. The relative molecular expression of NK cell was analyzed by FCM after anti-mouse immunofluorescent antibody staining. Similarly, the mice in the control group and the experimental group were randomly killed at day 14.5. In addition to recording the number of embryo implantation and embryo absorption, the endometrium of the embryo also needed to be peeled off, the development of the embryo was observed, and the crown-rump length of embryo and the weight of placenta were recorded respectively.

### Statistical analyses

The results of at least three independent experiments were analyzed using the Graphpad Prism 6 (GraphPad, CA, USA) statistical software package. When the data was normally distributed, the two groups were analyzed by paired or unpaired t-test, and one-way ANOV was conducted between multiple groups. When the data was non-normally distributed, the Mann-Whitney test, the Wilcoxon test or the Kruskal–Wallis test were generally used. Data were expressed as mean ± SEM, and considered statistically significant when *P* < 0.05.

## Results

### The level of autophagy in trophoblasts is decreased in RSA patients

**Fig. 1:**
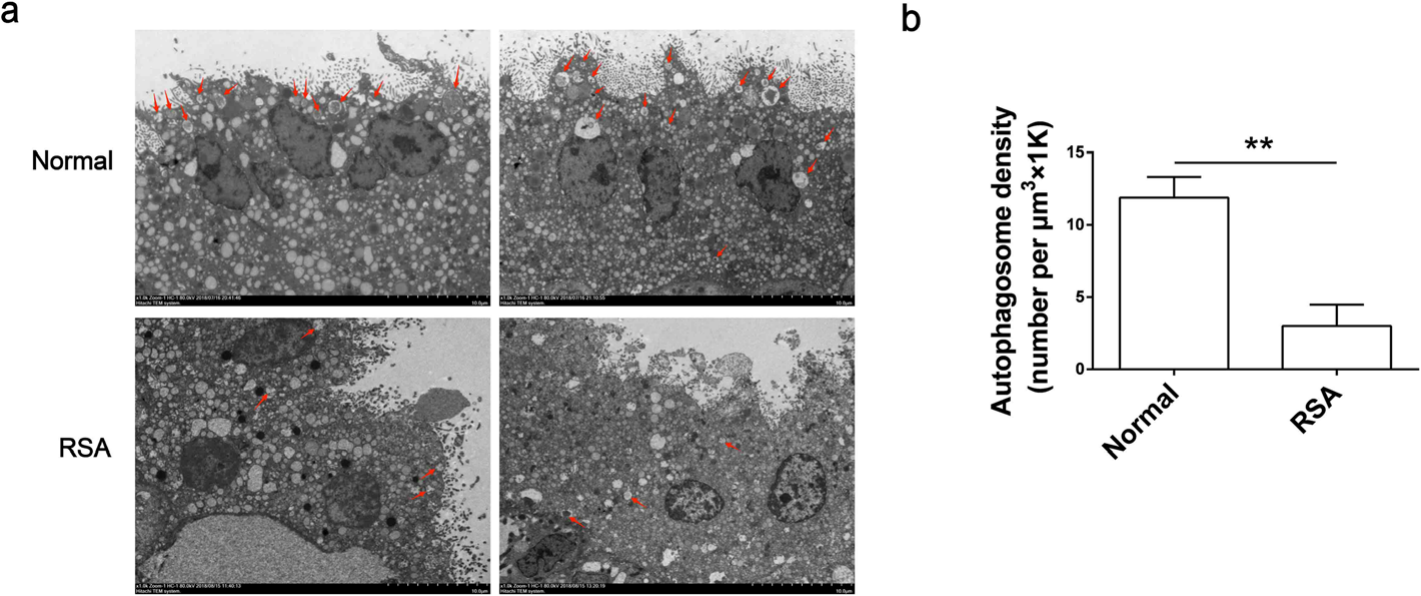
The level of autophagy in trophoblasts is decreased in RSA patients. **a**, **b** The number and distribution of autophagosomes in villi of normal women (*n* = 9) and RSA patients (*n* = 5) were detected by transmission electron microscopy. Original magnification: × 1.0 K. Bar = 10 μm. The red arrow indicates the autophagosome. Data are expressed as the mean ± SEM; unpaired t-test; **p < 0.01

To investigate the difference of autophagy level in trophoblasts between healthy pregnant women and RSA patients, we analyzed the number and distribution of autophagosome in villi by transmission electron microscopy (Fig. 1a). As shown in the picture, the villi of RSA patients displayed fewer double-wall membranes enclosed cytoplasmic vacuoles-namely autophagosome-compared with normal pregnancy patients (Fig. 1b). What’s more, the autophagosome in normal villus tissue is mainly distributed on microvillus surface, perhaps in syncytiotrophoblasts, while in RSA patients the distribution of autophagosome was in disorder. These results indicated that autophagy activity in trophoblasts was significantly lower in RSA patients compared with normal pregnant women.

### Autophagy in trophoblasts restricts the cytotoxicity of dNK cells

**Fig. 2:**
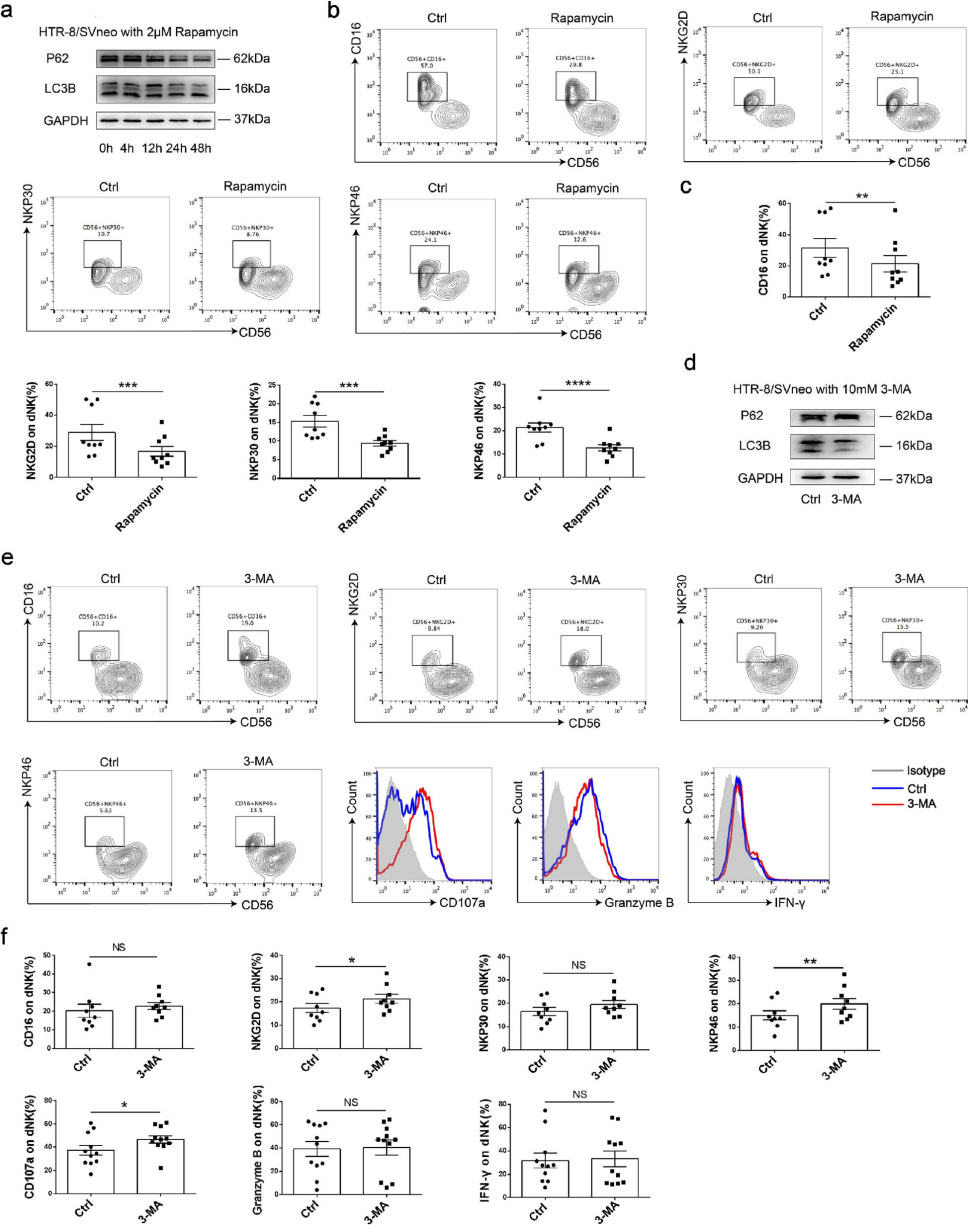
Autophagy in trophoblasts restricts the cytotoxicity of dNK cells. **a** The levels of autophagy-related proteins LC3B and P62 were detected by western blot. **b, c** HTR-8/SVneo cells were pretreated with rapamycin, then co-cultured with dNK (n = 9) cells for 48 h. The expression of CD16, NKG2D, NKP30 and NKP46 in dNK cells were detected by FCM. **d** The inhibitory effect of 3-MA on autophagy was verified by western blot. **e, f** HTR-8/SVneo cells were pretreated with 3-MA and then co-cultured with dNK (n = 9) cells for 48 h. The expression of CD16, NKG2D, NKP30, NKP46, CD107, IFN-γ and Granzyme B in dNK cells were detected by FCM. Data are expressed as the mean ± SEM; paired t-test or Kruskal-Wallis test; *p < 0.05, **p < 0.01, ***p < 0.001, ****p < 0.0001, NS: no significance

To confirm that autophagy in trophoblasts affects the phenotype and cytotoxicity of dNK cells, dNK cells were detected by flow cytometry (FCM) after co-culturing with trophoblasts pretreated with or without rapamycin. As shown, rapamycin significantly induced the autophagy of HTR-8/SVneo cells, especially for 48 h (Fig. 2a). Co-culturing with rapamycin pretreated HTR-8/SVneo cells suppressed the expressions of killer receptors (CD16, NKG2D, NKP30 and NKP46) in dNK cells (Fig. 2b, c). Consistent with this, dNK cells co-cultured with autophagy-inhibiting trophoblasts expressed significantly high levels of NKG2D, NKP46 and CD107a (Fig. 2e, f), while the difference between CD16, NKP30, Granzyme B and IFN-γ were not significant (data not shown) (Fig. 2f). These results suggest that autophagy in trophoblasts can restrict the cytotoxicity of dNK cells.

### Autophagy in trophoblasts inhibits NK cell killing activity by IGF-2

**Fig. 3:**
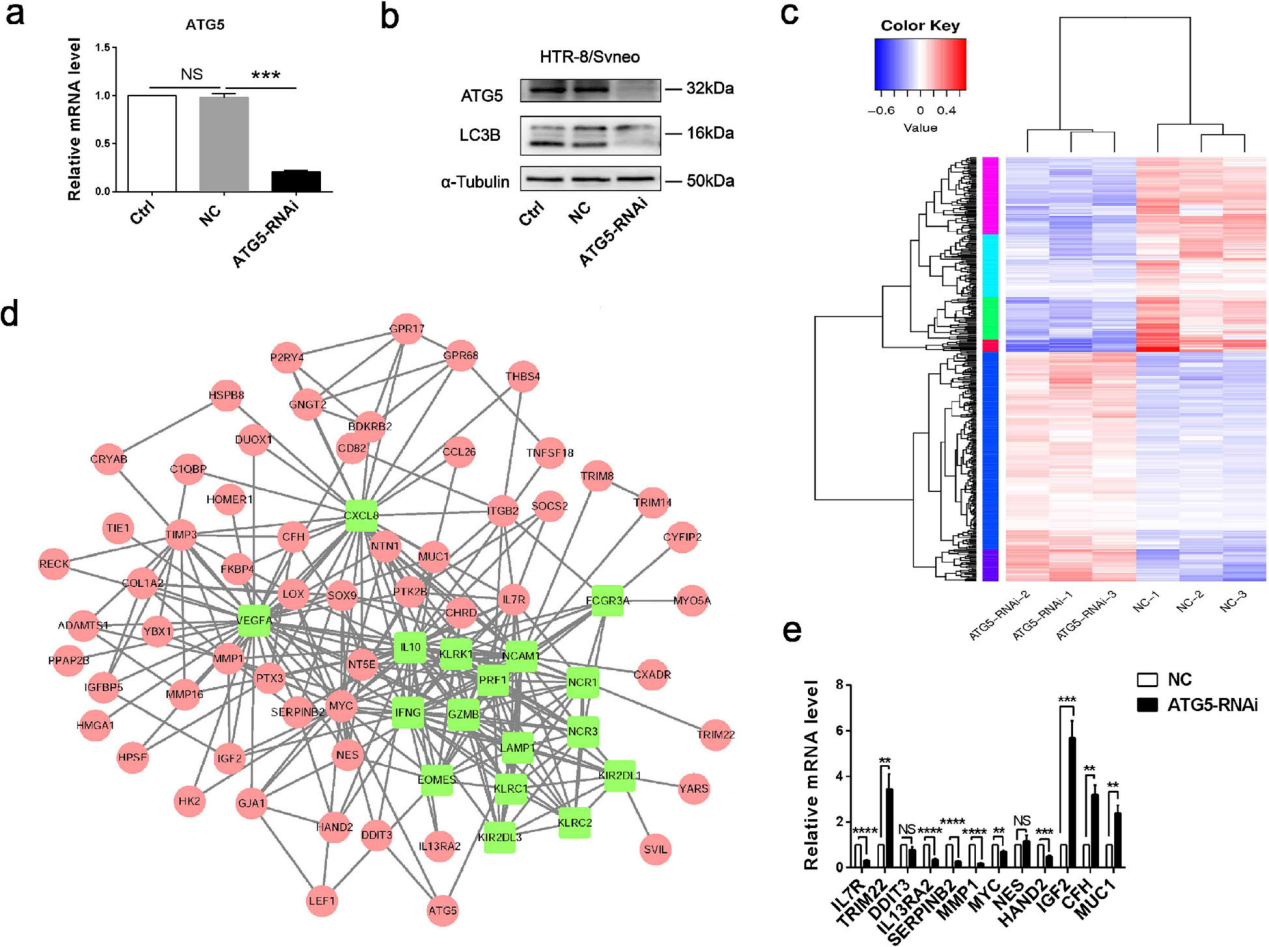
Lentiviral transfection with HTR-8/Svneo followed by RNA-seq. **a, b** Control、negative control and silencing efficiency of ATG5 were verified by qRT-PCR and western blot. Control(Ctrl), Negative control (NC). **c** The heat map of differential genes in control and *ATG5*-RNAi group. **d** The predicted networks of differential genes related to NK function. 12 differentially expressed molecules around NCR1 (NKP46) and IFNG (IFN-γ) were found. **e** Twelve related genes were verified in separate experiment. Date are expressed as the mean ± SEM; one-way ANOVA or paired t-test; **p < 0.01, ***p < 0.001, ****p < 0.0001, NS: no significance

Autophagy-related genes (*ATG*) play a regulatory role in autophagy, and their assembled complexes are activated and recruited to the membrane to initiate autophagy. To explore how autophagy in trophoblast regulating the phenotype of dNK cells, we inhibited autophagy in trophoblasts by silencing *ATG5*, and then performed RNA sequencing (RNA-seq) and bioinformatics analysis. The transfection efficiency of *ATG5*-RNAi and the autophagy inhibiting effect was verified separately (Fig. 3a, b). As shown in the figure, there were 388 differential expressed genes, of which 210 were up-regulated and 178 were down-regulated (Fig. 3c). According to the predicted network among differential genes and NK function-related genes, we found 12 differential molecules around NCR1 (NKP46) and IFNG (IFN-γ) (Fig. 3d). Four up-regulated genes included *TRIM22*, *IGF-2*, *CFH* and *MUC1*; and eight down-regulated genes included *IL7R*, *DDIT3*, *IL13RA2*, *SERPINB2*, *MMP1*, *MYC*, *NES* and *HAND2*. The mRNA expression of these 12 genes was verified in *ATG5*-silenced trophoblasts in separate experiments (Fig. 3e).

**Fig. 4:**
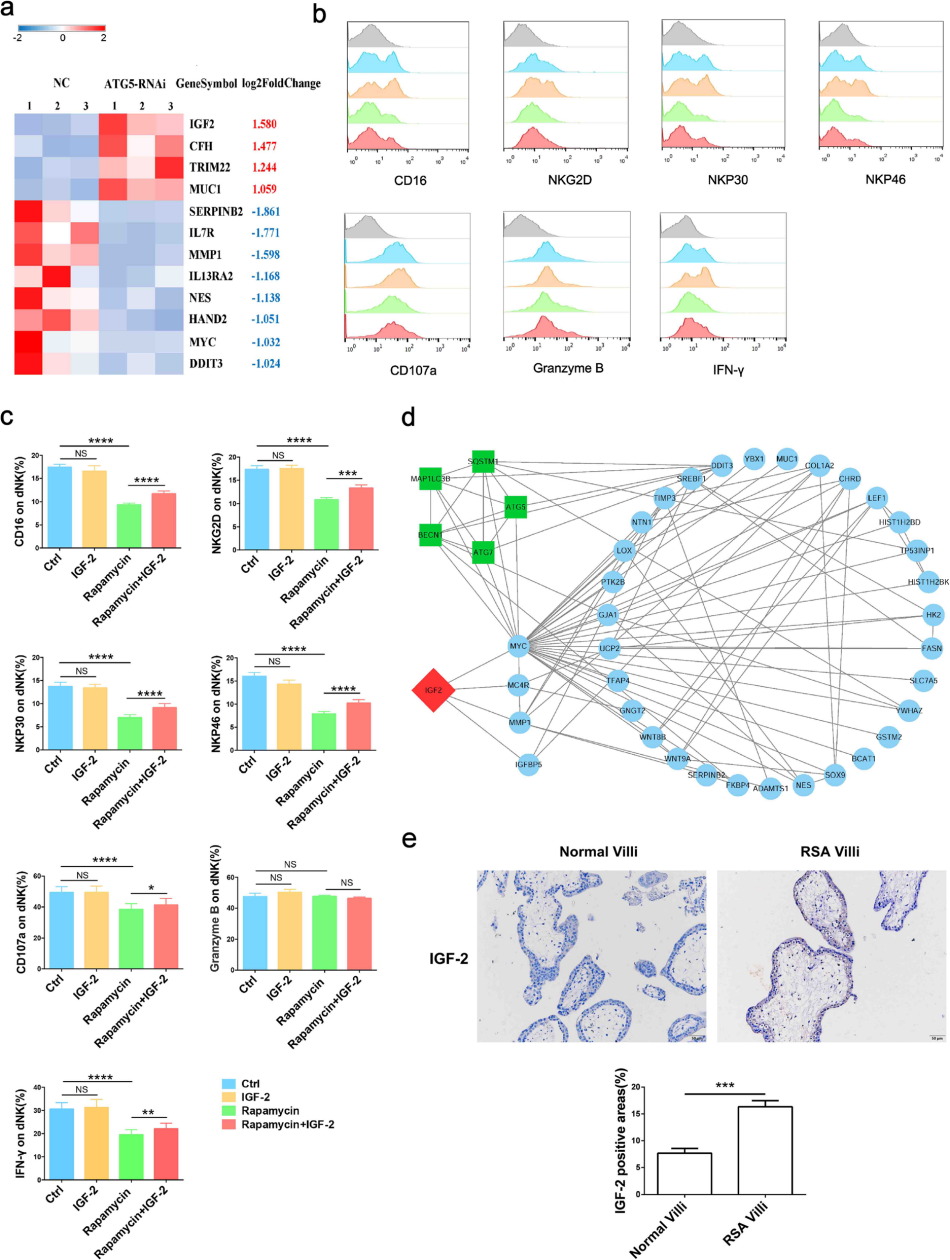
Autophagy in trophoblasts inhibits NK cell killing activity by IGF-2. **a** Heatmap of the differential genes associated with NCR1 and IFNG in control and *ATG5*-RNAi group. **b, c** dNK cells (*n* = 6) were co-cultured with trophoblasts pretreated by rapamycin or solvent control, with or without IGF-2 (50 ng/ul). The expressions of killing receptors in dNK cells were detected by FCM. **d**. Potential molecular pathways between autophagy and IGF-2 were predicted by bioinformatics analysis. **e** Immunohistochemistry analysis of IGF-2 expression in villi from normal pregnancy wowen (*n* = 5) and RSA patients (n = 5). Original magnification: × 200. The data are expressed as the mean ± SEM; one-way ANOVA; *p < 0.05, **p < 0.01, ***p < 0.001, ****p < 0.0001, NS: no significance

Among the 12 potential genes related to NK function, *IGF-2* was the most significantly upregulated in *ATG5*-RNAi group (Fig. 4a), which was also reported to be degraded by autophagy [25]. To verify whether autophagy in trophoblasts regulated the phenotype of dNK cells dependent on IGF-2, dNK cells were co-cultured with rapamycin pretreated trophoblasts, adding IGF-2 in the medium or not. As shown in the figure (Fig. 4b, c), the expression of killer receptors (CD16, NKG2D, NKP30, NKP46, CD107a and IFN-γ) in rapamycin-treated group were decreased significantly, which was consistent with our previous results. Furthermore, the addition of IGF-2 could partially reverse the inhibitory effect of trophoblast autophagy on the killing activity of dNK cells. IGF-2 addition in the co-culture medium had no effect on dNK cells, which might imply that trophoblasts could secret sufficient IGF-2 in normal condition. Taken together, these data validate that autophagy in trophoblasts may suppress NK cytotoxicity by decreasing the IGF-2 secretion. And the potential pathways in which autophagy regulate the expression of IGF-2 were predicted by the bioinformatics analysis (Fig. 4d). In vivo trials further verified that the villi of RSA patients showed more IGF-2 than those of normal pregnancy patients (Fig. 4e).

### Autophagy in trophoblasts promotes self-invasion via the PEG10

**Fig. 5:**
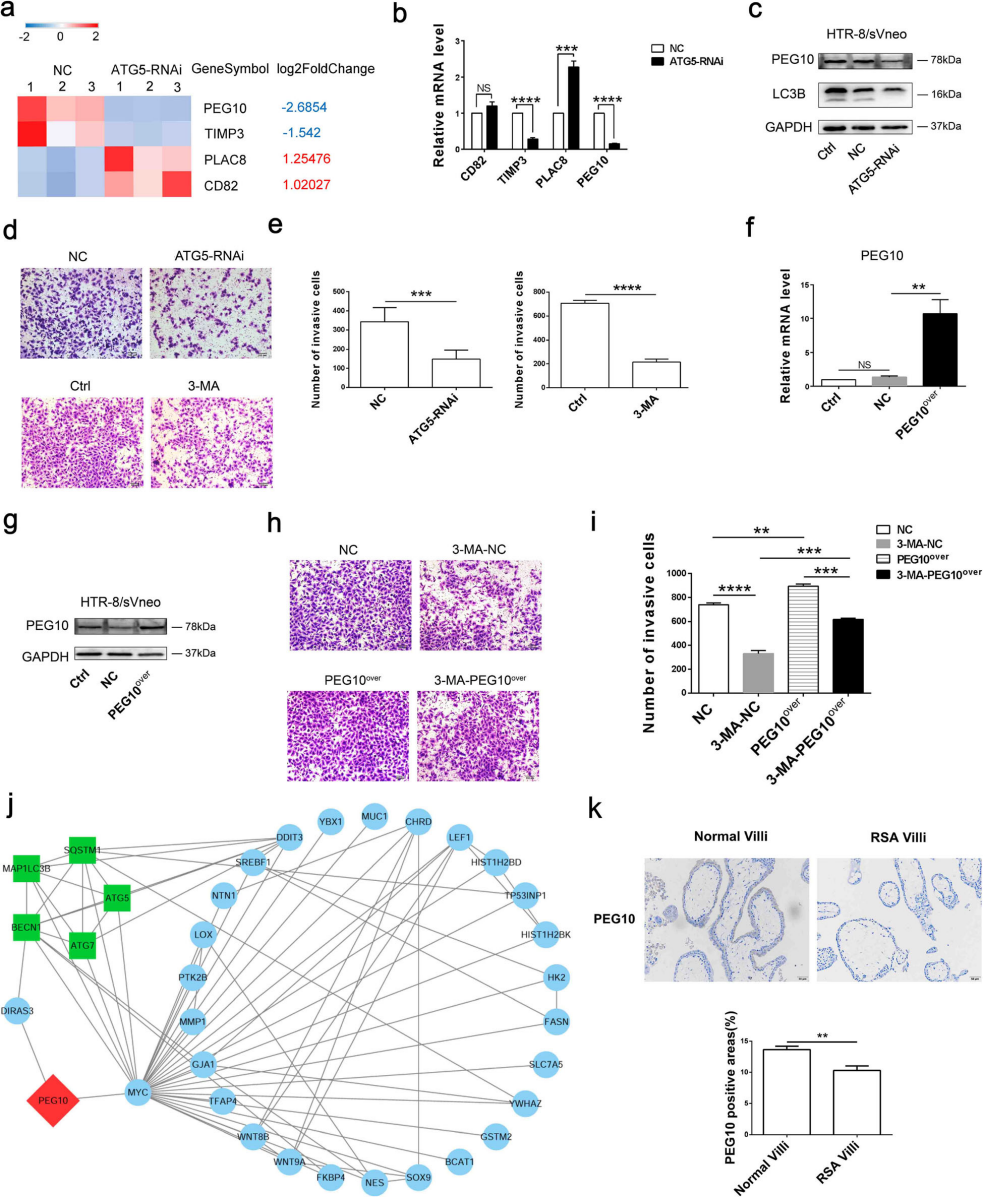
Autophagy in trophoblast promotes the invasion of itself via the PEG10. **a** Heatmap of the differential genes associated with trophoblast invasion in control and *ATG5*-RNAi group. **b** The four screened genes were identified by qRT-PCR. **c** PEG10 expression was verified by western blot. **d, e** The invasion of trophoblasts that transfected with negative control lentivirus, ATG5 silencing virus, treated with 3-MA or control was detected by transwell assay. Scale bar: 100 μm. **f, g** PEG10 expression levels in trophoblast transfected with PEG10 overexpression lentivirus, negative control lentivirus or the control group were verified by western blot and qRT-PCR. **h, i** The invasion of trophoblasts in NC group, 3-MA-NC group, PEG10^over^ group, 3-MA-PEG10^over^ group was detected by transwell assay. Scale bar: 100 μm. **j** Potential molecular pathways between autophagy and PEG10 were predicted by bioinformatics analysis. **k** PEG10 expression in villi of normal pregnancy women (n = 5) and RSA parients (n = 5) by immunohistochemistry. Original magnification:× 200. The data are expressed as the mean ± SEM; one-way ANOVA, paired t-test; *p < 0.05, **p < 0.01, ***p < 0.001, ****p < 0.0001, NS: no significance

To explore the effect of autophagy on biological behaviors of trophoblasts itself, we performed a predicted network analysis among differential genes (Fig. 3c) and trophoblast invasion-related genes. Only four overlapping molecules (*TIMP3*, *PLAC8*, *CD8*2 and *PEG10*) were found (Fig. 5a). In the next verification experiment, we discovered that *PLAC8*, *PEG10* and *TIMP3* were changed in accordance with RNA-sequencing results, while *CD82* was not significantly different (Fig. 5b). We further focused on PEG10 as the potential target through which autophagy might regulate the invasion in trophoblasts. *PEG10* is an important imprinting gene for paternal expression and maternal imprinting. It is found to be located in 7q21, which is expressed in both adult and embryonic tissues, but is significantly expressed in the placenta [26]. It was verified that silencing *ATG5* could effectively decreased the expression of PEG10 in trophoblasts (Fig. 5c), and 3-MA could also inhibit PEG10 mRNA and protein levels (Figure S1). When autophagy was inhibited by 3-MA or silencing *ATG5*, the invasion of HTR-8/SVneo was reduced obviously (Fig. 5d, e).

To clarify whether autophagy in trophoblasts affects self-invasion via the PEG10, we further transfected HTR-8/SVneo with PEG10 overexpression lentivirus and verified the efficiency of PEG10 expression in mRNA and protein level. (Fig. 5f, g). Then, we divided HTR-8/SVneo into 4 groups: NC group, 3-MA-NC group, PEG10^over^ group, 3-MA-PEG10^over^ group. The results showed that 3-MA could significantly inhibit the invasion of trophoblasts, and this effect was partly abolished by PEG10 (Fig. 5h). Actually, compared with PEG10 overexpressed group, the invasion ability was also suppressed in 3-MA-PEG10^over^ group (Fig. 5i), suggesting that it may be a dual regulation loop between autophagy and PEG10, autophagy in trophoblast promotes the invasion of itself via the PEG10. To identify possible downstream signaling molecules, we found out 28 differential genes in RNA sequence associated with autophagy and PEG10, in which *MYC* may play a critical role (Fig. 5j). In vivo, the expression of PEG10 in villi from RSA patients were significantly lower than that villi from normal pregnancy women (Fig. 5k).

### dNK cell educated by autophagy-inducing trophoblasts regulates the proliferation and invasion of trophoblasts

**Fig. 6:**
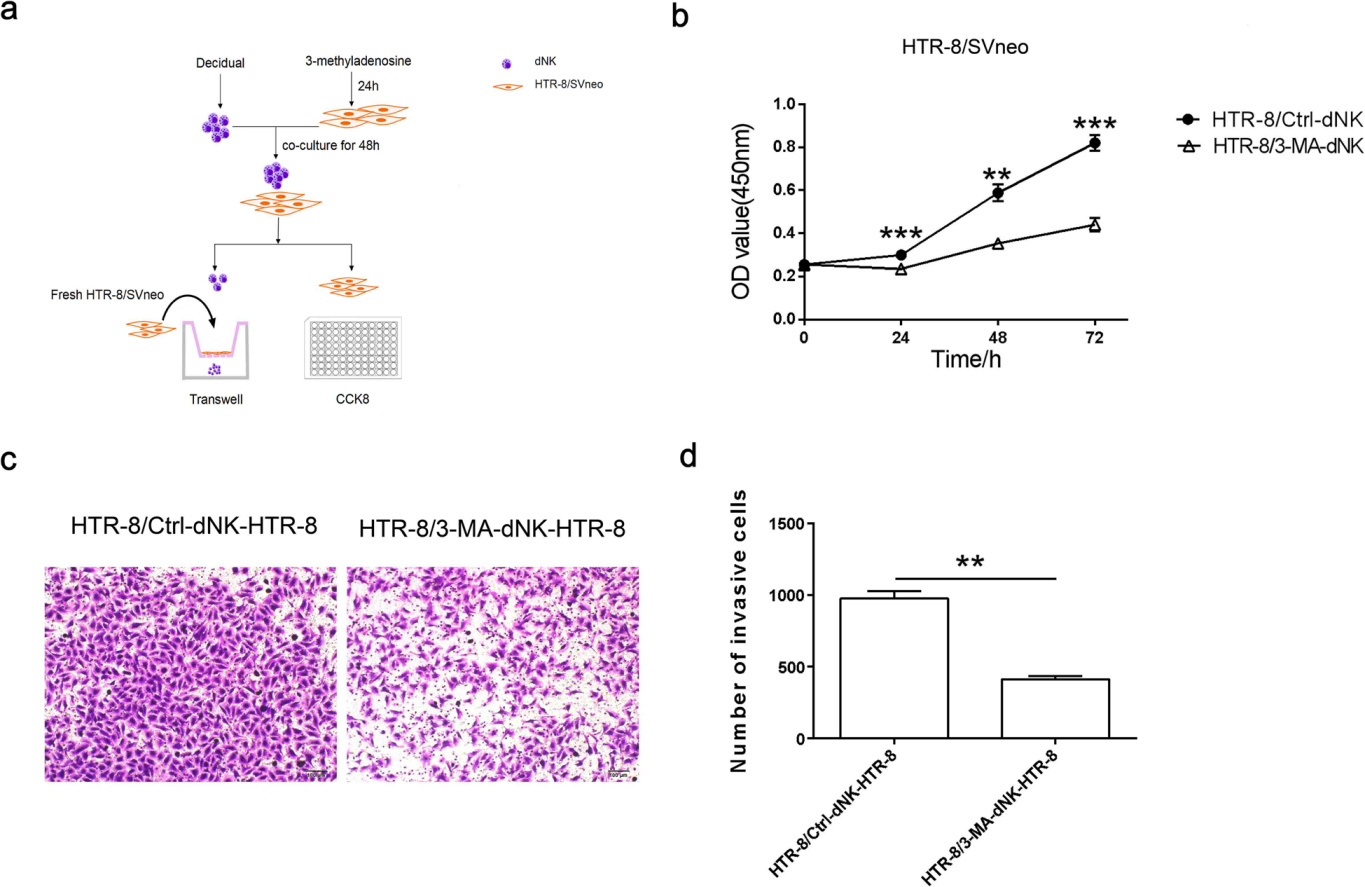
dNK cell educated by autophagy-inducing trophoblasts affects the proliferation and invasion of trophoblasts. **a** Schematic process of cell treatment. dNK cells were co-cultured with 3-MA treated trophoblast for 48 h. Then, the trophoblasts were collected to detect the viability by CCK8 and the dNK cells were collected to co-culture with fresh trophoblasts indirectly. The invasion of these fresh trophoblasts was measured by transwell assay. **b**. Cell viability of trophoblasts was detected by CCK8. **c, d** The invasion of trophoblasts was detected by transwell assay. Scale bar: 100 μm. The data are expressed as the mean ± SEM; paired t-test; **p < 0.01; ***p < 0 .001

To explore whether dNK cells educated by trophoblasts could affect the behavior of trophoblasts in return, we collected dNK cells co-cultured with pretreated trophoblast and co-cultured them with fresh trophoblasts indirectly (Fig. 6a). The viability of pretreated-trophoblasts was detected by CCK8 after co-cultured with dNK cells. As is shown in the figure, the viability in 3-MA treated group was decreased significantly (Fig. 6b). And the invasion of trophoblasts co-cultured with dNK cells in 3-MA group was also decreased (Fig. 6c, d). Taken together, we conclude that autophagy-inhibition in trophoblasts impairs the effect of dNK cells on promoting proliferation and invasion.

### Inhibition of autophagy in trophoblasts increases dNK cell killing activity and embryo absorption rate in vivo

**Fig. 7:**
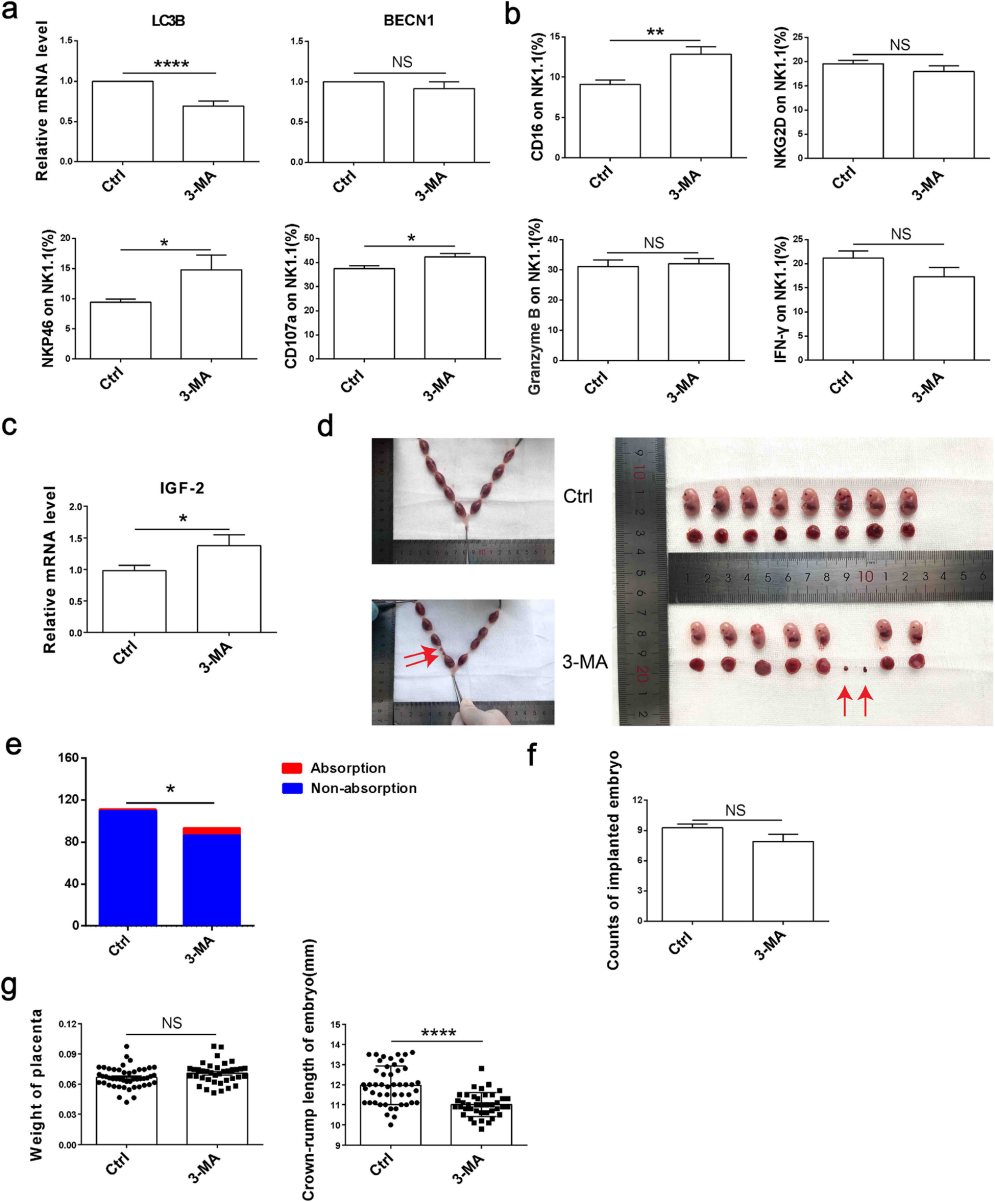
Inhibition of trophoblasts autophagy increases dNK cell killing activity and embryo absorption rate in vivo**. a** The mRNA expression of autophagy-associated molecules (LC3B, Beclin) was detected by qRT-PCR in placental. **b** At 8.5 days of pregnancy, the expression of NK killer receptors in the uterus were detected by FCM (Ctrl, *n* = 6, 3-MA, n = 6). **c** The mRNA expression of IGF-2 in placenta of mice was detected by qRT-PCR (Ctrl, n = 6; 3-MA, n = 6). **d, e** Embryo absorption rate of control group and 3-MA group (Ctrl, *n* = 12; 3-MA, *n* = 11). **f** The number of embryo implantations (Ctrl, n = 12; 3-MA, n = 12). **g** The weight of placenta and the embryo crown-rump length in both groups (Ctrl, n = 6; 3-MA, n = 6). The data are expressed as the mean ± SEM; unpaired t-test, Mann–Whitney, Chi-square test; *p < 0.05, **p < 0.01, ***p < 0.001, ****p < 0.0001, NS: no significance

To verify the effect of trophoblasts autophagy on uterine dNK cells and embryo absorptivity in vivo, pregnant C57BL6J mice model was established. 3-MA or saline were given by intraperitoneal injection at day 0, day 4.5 and day 10.5 of gestation. In comparison with control group, placental from 3-MA-treated pregnant mice had a low level of LC3B, proving that trophoblast autophagy was inhibited effectively in 3-MA group (Fig. 7a). The killing activity of mice uterine dNK cells were detected at 8.5 days of gestation. FCM results indicated that the expression of CD16, NKP46 and CD107a of dNK cells in 3-MA group were higher than the control group, but NKG2D, Granzyme B and IFN-γ had no significant change (data not shown) (Fig. 7b). Consistently, IGF-2 was increased in the placenta of the 3-MA group (Fig. 7c).

To investigate the influence of trophoblasts autophagy inhibition on pregnancy outcome, we evaluated the abortion rate, placenta weight, and the crown-rump length of embryo at 14.5 days of gestation. No significant difference was detected in the number of implantation after 3-MA treatment, but the absorption rate in 3-MA group was increased (Fig. 7d-f). And compared with the control group, the crown-rump length of embryo in the 3-MA group was decreased, while the placental weight did not change (Fig. 7g). In conclusion, our study confirms that inhibition of autophagy in trophoblast promotes the killing activity of dNK cells and increases fetal loss in mice.

## Discussion

Autophagy is a non-apoptotic form of over-activated programmed cell death [27, 28]. During the process of autophagy, both autophagy-related genes (ATG) and microtubule-associated protein 1 light chain 3 (MAP1LC3, commonly known as LC3) are involved in the development and maturation of autophagosome. Especially, ATG5 participates in the formation of the complex ATG12-ATG16L1, and then recruits LC3 on the phage membrane and promotes the processing of LC3 [29, 30]. Autophagy plays an indispensable role in early embryonic development, which is often associated with abortion, preeclampsia, intrauterine growth restriction [31–33]. In this study, we found that the level of autophagy in villi of RSA patients was significantly lower than that of elective pregnancy termination patients. Here, we demonstrate that the reduction of autophagy in trophoblasts increases the killing activity of dNK cells through IGF-2, and impairs PEG10-mediated trophoblasts invasion.

IGF-2 is a multifunctional cell proliferation regulation factor, which plays an important role in cell differentiation, embryo growth and development, and tumor cell proliferation [34, 35]. The effect of IGF-2 is mainly mediated by the insulin receptor (IR); type1 IGF receptor (IGF-1R); type2 IGF receptor (IGF-2R) [36]. For example, the binding of IGF-2 and IGF-1R can activate the tyrosine kinase domain, triggering mitogen-activated protein kinase (MAPK)/extracellular signal-regulated kinases (ERK) and phosphoinositide 3-kinase (PI3K)/AKT (protein kinase B) to promote anti-apoptotic effect, which in turn triggers the effect on cell proliferation by the mammalian target of rapamycin (mTOR) pathway [37, 38]. At present, little is known about the regulatory effect of IGF-2 on NK cell function. IGF-2 may regulate the killing effect of NK cells through the START3 (signal transducer and activator of transcription 3) signaling pathway. Our previous research has found that the signal START3 plays an important role in NK cytotoxicity or functional regulation [11]. IGF-2, which is present in tumor cells, can be secreted by activated STAT3 to impair the antitumor efficacy of anti-IGF-2 therapy [39]. Therefore, we speculate that IGF-2 may affect NK cell killing activity in an indirect way. In our current research, we found that IGF-2 could be induced by autophagy-inhibited trophoblast cells. And whether autophagy in trophoblast cells regulates NK cell killing activity by IGF-2 should to be further studied.

After co-cultured with 3-MA-pretreated HTR-8/SVneo cells, the killing activity of dNK cells was increased significantly. Similar results were obtained in vivo. Interestingly, supplementation with IGF-2 partially reversed the effect of autophagy in trophoblast cells on the killing receptor of NK cells, suggesting that trophoblasts autophagy inhibits NK cell killing activity by IGF-2. In fact, the specific regulation of IGF-2 by trophoblasts autophagy is largely unknown. Although this study was the first to discover that autophagy in trophoblast cells could regulate NK cell cytotoxicity through IGF-2, we were unable to further validate it in vivo due to the lack of ATG5 knockout mice. Fortunately, the protein interaction between IGF-2 and autophagy-related genes were analyzed based on our sequencing results. It was found that autophagy-related genes were associated with IGF-2 through 35 differential genes. This provides a reliable basis for the next experiments.

In addition, autophagy is also associated with cell invasion. We confirmed that 3-MA caused a significant reduction of invasiveness in trophoblast cells, which is consistent with previous research findings [40]. According to bioinformatics analysis, we found the most different gene PEG10. As reported, PEG10 promotes trophoblasts invasion by TIMP-1, and PEG-10 knockout may cause placental defects and early embryonic lethality in mice [41, 42]. This suggests that the decrease of PEG10 gene may be a cause of recurrent abortion. PEG10 is also considered as a cancer imprinting gene. Several scholars have pointed out that E2F1-mediated PEG10 overexpression can promote the proliferation, invasion and migration of tumor cells (pancreatic cancer, liver cancer and prostate cancer, etc.) [43–45]. Li et al. demonstrate that curcumin inhibits SIAH1-mediated apoptosis via the miR-491/PEG10 pathway, leading to stagnant cancer cell growth [46]. In this study, our findings indicate that autophagy inhibition decreased the level of PEG-10 in trophoblast cells. Subsequently, we validated that autophagy could positively regulate the invasive function of trophoblast cells via the PEG10. Unfortunately, the regulatory mechanism between trophoblasts autophagy and PEG10 in this experiment has not been deeply involved, and we can further study it based on existing clues.

There is a correlation between HTR-8/SVneo and educated dNK. Cichocki et al have reported that there is a clear immunological memory characteristic of mature natural killer cells in response to chronic human cytomegalovirus (HCMV) [47]. Ghofrani et al revealed that “memory” NK cells can also be induced by cytokines (such as IL-12, IL-15 and IL-18, [48]). Gamliel et al. disclosed that is mainly restricted to NKG2Chi decidua “memory” NK cells (called PTdNKs), high expression receptors NKG2C and LILRB1, supporting repeated pregnancy [49]. Unlike HCMV and cytokine-induced “memory” NK cells, PTdNKs not only secrete high amounts of IFN-γ, but also uniquely exhibit higher secretion of VEGFα, promoting angiogenesis and decidua maintenance at implantation sites. In this research, it is confirmed that there is a negative feedback regulation loop between the HTR-8/SVneo and educated dNK. Based on the above evidence, dNK cell educated by autophagy-inducing trophoblasts may have the different secretion of cytokines, which may further affect the invasion ability of trophoblasts,

However, the regulation mechanism of “memory” NK cells require further work.

## Conclusions

**Fig. 8:**
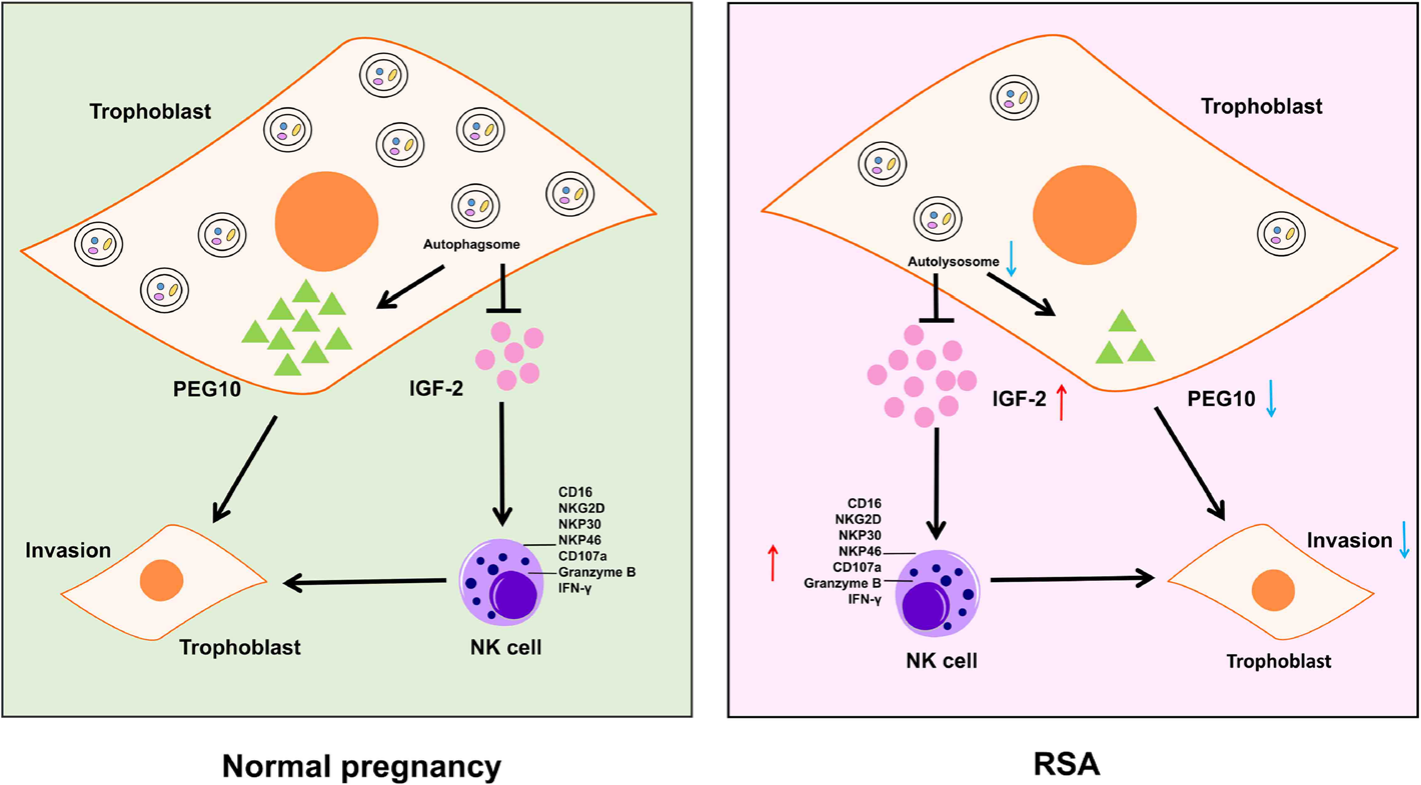
Schematic roles of trophoblasts autophagy on dNK cytotoxicity and trophoblast invasion in early pregnancy. Autophagy in trophoblast cell is decreased in RSA patients. On the one hand, low level of autophagy in trophoblast leads to the increase of IGF-2 secretion, which further leads to the high killing activity of NK cells attacking the normal cells; on the other hand, autophagy suppression of trophoblast cells decreases the PEG10, which reduces the invasion of trophoblasts. In addition, dNK cells educated by trophoblasts can affect the behavior of trophoblasts in return. Autophagy depression in trophoblast suppresses the effect of dNK cells on promoting proliferation and invasion. As a result, the impairment of autophagy in trophoblast may leads to recurrent spontaneous abortion

Collectively, as shown (Fig. 8), we have demonstrated that autophagy suppression of trophoblast cells induces RSA through IGF-2 secretion and PEG10 reduction. On the one hand, high levels of IGF-2 leads to NK cells differentiation, and these NK cells with high killing activity attacked normal cells at the maternal-fetal interface. On the other hand, autophagy suppression of trophoblast cells decreases the PEG10, which reduces the invasive function of trophoblasts and leads to pregnancy maintain failure. In addition, dysfunctional “memory” NK cells in the first pregnancy will continue to affect newly formed trophoblasts, and these processes will form a vicious positive feedback cycle that will ultimately accelerate the progression of RSA. Based on this research, future RSA treatments can be combined with autophagy inducers. Previous reports have suggested the idea of targeted autophagy for cancer treatment [50]. For example, rapamycin is both an immunosuppressant and an autophagy inducer. It may have therapeutic value for immune abortion with low level of autophagy in trophoblast. Simultaneously, IGF-2 inhibitors can alleviate the NK cell toxicity mediated by IGF-2, and have potential value in the treatment of diseases associated with NK cytotoxicity.

## Supplementary information

**Additional file 1: Supplementary Figure 1.** 3-MA treatment of HTR-8/SVneo decreases the expression of PEG10. a,b After HTR-8/SVneo was treated with 3-MA, the mRNA and protein levels of PEG10 were detected. The data are expressed as the mean ± SEM; paired t-test; *p < 0.05.

## Data Availability

The datasets involved in our study are available on reasonable request.

## Abbreviations

dNK: Decidual natural killer cells
RSA: Recurrent spontaneous abortion
3-MA: 3-Methyladenosine
IGF-2: Insulin-like growth factor-2
PEG10: Paternally Expressed Gene 10
CCK-8: Cell-counting kit-8
FCM: Flow cytometry
DIC: Decidual immune cell
ATG: Autophagy-related genes
MAP1LC3: Microtubule-associated protein 1 light chain 3
MAPK: Mitogen-activated protein kinase
ERK: Extracellular signal-regulated kinases
PI3K: Phosphoinositide 3-kinase
START3: Signal transducer and activator of transcription 3
mTOR: Mammalian target of rapamycin
